# Associations of epidemiologic risk factors with *Fusobacterium nucleatum* and bacterial alpha diversity in the colorectal tumor-associated microbiota

**DOI:** 10.1007/s10552-026-02133-4

**Published:** 2026-02-10

**Authors:** Courtney M. Hill, Rachel C. Malen, Adriana M. Reedy, Orsalem Kahsai, Keith Curtis, Ningxin Ma, Timothy W. Randolph, Jing Ma, Claire E. Thomas, Shuji Ogino, John D. Potter, Daniel D. Buchanan, Polly A. Newcomb, Meredith A. J. Hullar, Amanda I. Phipps

**Affiliations:** 1 Department of Epidemiology, University of Washington, Seattle, WA 98105 USA; 2 Fred Hutchinson Cancer Center, Seattle, WA 98109 USA; 3 Program in MPE Molecular Pathological Epidemiology, Department of Pathology, Brigham and Women’s Hospital, Harvard Medical School, Boston, MA 02215 USA; 4 Department of Epidemiology, Harvard T.H. Chan School of Public Health, Boston, MA 02215 USA; 5 Cancer Immunology Program, Dana-Farber Harvard Cancer Center, Boston, MA 02115 USA; 6 Broad Institute of MIT and Harvard, Cambridge, MA 02114 USA; 7 Tokyo Medical and Dental University (Institute of Science Tokyo), Tokyo, Japan; 8 Centre for Public Health Research, Massey University, Wellington, New Zealand; 9 Colorectal Oncogenomics Group, Department of Clinical Pathology, Melbourne Medical School, The University of Melbourne, Parkville, Australia; 10 Collaborative Centre for Genomic Cancer Medicine, The University of Melbourne, Victorian Comprehensive Cancer Centre, Parkville, Australia; 11 Genomic Medicine and Family Cancer Clinic, The Royal Melbourne Hospital, Parkville, Australia

**Keywords:** Intratumoral microbiome, Fusobacterium, 16S rRNA gene sequencing, Bacterial diversity, CRC, SCCFR, Diet, Physical activity, Socioeconomic status, ddPCR

## Abstract

**Background:**

Aspects of the gut microbiome, including presence of specific bacterial species and overall community structure, have been linked to the etiology and prognosis of colorectal cancer (CRC). Less is known about the epidemiologic risk factors that are associated with the composition of the microbiota in invasive colorectal tumors.

**Methods:**

Using tumor and paired normal colorectal tissue samples from a subset of participants in the population-based Seattle Colon Cancer Family Registry, we compared the presence of *Fusobacterium nucleatum (F. nucleatum)* (*n* = 898) measured via droplet digital PCR and alpha diversity (Shannon index) (*n* = 611) measured via 16S rRNA gene sequencing in colorectal tissue across demographics, health behaviors, and neighborhood socioeconomic status (nSES).

**Results:**

Normalized counts of *F. nucleatum* were consistently higher in tumor tissue than in patient-matched normal tissue across all risk factors, while alpha diversity was lower. Female sex was associated with high presence and enrichment of *F. nucleatum* in tumor tissue (odds ratio [OR] 1.61; 95% confidence interval [CI] 1.02, 2.54 and OR 1.58, 95% CI 1.10, 2.27, respectively). Relative to those aged 40–49 years, the youngest age group (< 40 years) had lower alpha diversity in tumor tissue (OR for highest vs. lowest tertile: 0.33; 95% 0.13, 0.83). Other factors, including diet, were not related to *F. nucleatum* presence or tumor tissue alpha diversity.

**Conclusion:**

By uncovering epidemiologic risk factors for *F. nucleatum* presence and bacterial diversity in the intratumoral microbiota, this work informs our understanding of associations of the gut microbiota with CRC etiology and outcomes.

**Supplementary Information:**

The online version contains supplementary material available at 10.1007/s10552-026-02133-4.

## Introduction

Colorectal cancer (CRC) [1, 2], the third most commonly diagnosed cancer and the second leading cause of cancer-related mortality [3], is a multi-factorial disease. CRC etiology is influenced by a combination of genetic, demographic, behavioral, and environmental factors. In addition to established risk factors, like diet and physical inactivity [4], characteristics of the gut microbiome are increasingly recognized to play a role in CRC development and progression. In particular, presence of bacterial species *Fusobacterium nucleatum (F. nucleatum)* in the gut microbiota has been implicated in CRC etiology, [5–9] and the presence of these bacteria in the intratumoral microbiota has been associated with CRC survival [8]. Biologic mechanisms underlying these associations involve expression of transcription factors, oncogenes, and inflammatory genes, and modulation of the tumor immune microenvironment [10, 11].

In addition to the presence and abundance of specific bacteria (e.g., *F. nucleatum*), the overall community structure of the gut microbiota and the intratumoral microbiota specifically may also have a role in CRC. A small number of studies have indicated that gut microbial diversity is lower in individuals with adenomas than in healthy controls and is lowest in CRC patients [1, 12, 13].

Emerging research is aimed at evaluating a potential link between established risk factors for CRC and the tumor-associated microbiota. One area of work has focused on diet as a potential predictor of *F. nucleatum* presence in the gut, where studies have suggested that inflammatory diets are associated with higher risk of *F. nucleatum*-positive colorectal tumors [14] and fiber-rich diets are associated with lower risk of *F. nucleatum*-positive colorectal tumors [15]. Other work has explored the relationship between age at CRC diagnosis and bacterial diversity, suggesting that tumor bacterial diversity differs across young-onset (< 50 years) and average onset cancers and stage of diagnosis, although the direction of the relationship is disputed [16, 17].

Given that aspects of the intratumoral microbiota are associated with CRC etiology and poorer CRC survival, it is important to identify epidemiologic risk factors that are associated with composition of the intratumoral microbiota. However, beyond diet and age, other epidemiologic risk factors have scarcely been considered in relation to either the presence of specific bacterial species or the overall bacterial diversity in the tumor microbiota.

Using data from a large, population-based study, we examined the relationships of several established epidemiologic risk factors for CRC with *F. nucleatum* presence and enrichment and with bacterial alpha diversity in CRC tissue.

## Methods

### Study Population

The Puget Sound Colorectal Cancer Cohort (PSCCC) is primarily comprised of study participants from the Seattle site of the Colon Cancer Family Registry (SCCFR) [18] and includes a subset of additional participants from a parallel extension study [19] as previously described. Participants for both studies included in this analysis from the PSCCC were identified through the Surveillance, Epidemiology, and End Results (SEER) cancer registry serving the Seattle-Puget Sound region. For the SCCFR, we enrolled study participants who were diagnosed with incident invasive CRC between 1998 and 2007 while residing in western Washington State; in Phase I (1998–2002), we enrolled those who were diagnosed at ages 20–74 years while residing in a three county region, and in Phase II (2002–2007) we restricted enrollment to those diagnosed at ages 18–49 years but expanded to a 13-county catchment area. Enrollment for the extension study included individuals of female sex who were diagnosed with incident invasive CRC between 1998 and 2002 while residing in the broader 13-county region. Inclusion in the PSCCC was limited to English-speakers with available telephone numbers who completed a baseline epidemiologic questionnaire. This baseline questionnaire included information regarding demographic factors, as well as a range of factors relating to lifestyle, medical history, and family history of cancer. The present analysis was further restricted to individuals for whom microbial DNA could be extracted from matched diagnostic tumor and normal colorectal tissue for analyses of *F. nucleatum* (*n* = 898) and *16S rRNA* gene sequencing for alpha diversity (*n* = 611) (**Supplementary Fig. 1**).

All study participants included in the present analysis provided informed consent for the collection and analysis of their study materials. This study was approved by the Institutional Review Board of the Fred Hutchinson Cancer Center (IR#8650).

### Exposures: epidemiologic risk factors

We examined several established epidemiologic CRC risk factors as exposures in relation to microbial characteristics, including demographic factors, health behaviors and personal history, and neighborhood socioeconomic status (nSES). Study variables were parametrized as shown in Table 1.

Information on demographic factors was available from SEER records (i.e., age at diagnosis, sex) and via self-report on a study baseline questionnaire (i.e., race, education). *Health behaviors and personal history* exposures were also ascertained from a baseline questionnaire and included variables for the self-reported intake of vegetables, fruit, red meat, and alcohol, cigarette use, physical activity, non-steroidal anti-inflammatory drug (NSAID) use, CRC family history, and body mass index (BMI). The study questionnaire was completed by telephone interview and participants were asked about average dietary intake from a time approximately two years prior to CRC diagnosis. For vegetables and fruits, intake was categorized as < 1 serving per day (low), 1 serving per day (medium), and > 1 than one serving per day (high), based on the observed distribution of participant responses. Red meat intake was categorized into tertiles: < 0.28 servings/day (low), 0.28–0.57 servings/day (medium), and > 0.57 servings/day (high). We used the most recent decade of life to categorize alcohol intake, and categories were no drinks per week (none), 1–6 drink(s) per week (medium), and ≥ 7 drinks per week (high).

During the interview, participants were also asked to self-report detailed information on their recreational physical activity during defined age periods prior to diagnosis: ages 20–29, 30–49, and 50 + years [20]. Questions covered different modes of activities (e.g., walking, jogging, running, bicycling, swimming, soccer, tennis, basketball, calisthenics), and the usual duration and frequency of each activity. Evaluation was limited to activities in which the patients were engaged for at least 30 min per week, for at least 3 continuous months. Standard metabolic equivalent of task score (METS) values were assigned to each activity [21] and multiplied by the number of hours per week engaged in that activity to derive METS-hours per week (METS-h/week). Physical activity during the age period of a participant’s CRC diagnosis (i.e., 20–29, 30–49, or 50 + years) was then summarized as average METS-h/week. For the present analysis, we categorized this variable as < 10 (low), 10–50 (medium), and > 50 METS-h/week (high). Cigarette smoking history and regular use of non-steroidal anti-inflammatory drugs (NSAIDs) use were categorized as ever or never.

nSES was assessed on the Census Block Group level using participant’s residential address at CRC diagnosis as previously described [22]. Briefly, nSES was assessed as a composite variable using six data elements from the 5-year American Community Survey data estimates: median household income, median housing unit value, percentage of households earning income from investments, percentage of persons aged ≥ 25 years who have completed high school, percentage of persons aged ≥ 25 years who have completed a college degree, and percentage of persons aged ≥ 16 years in a managerial or professional occupation. Each element was z-transformed and then summed resulting in a composite score that ranged from -15 to 15 and was divided into quartiles: [-15,-3.29], (-3.29,0.0697], (0.0697,3.72], and (3.72,15]. In the composite variable, lower scores indicated lower nSES.

### Study outcomes: *F. nucleatum* enrichment and presence and tumor tissue alpha diversity

#### Tissue collection and bacterial DNA extraction

Formalin-fixed paraffin-embedded (FFPE) diagnostic tumor tissue specimens were obtained from treating institutions for consenting study participants, as were FFPE tissue specimens from paired normal colorectal tissues from the same patient. Tissue samples were all processed into FFPE blocks shortly following diagnosis at time of procedure (biopsy or resection) by treating institutions and were procured by the CCFR shortly after, following completion of the baseline questionnaire and receipt of signed informed consent for tissue collection (1998–2007). FFPE blocks were sectioned primarily at Fred Hutch and stored as slides (typical thickness of 5 or 7 microns but ranging from 4 to 10 microns) or scrolls in tubes (10 microns). Many institutions did not request their blocks returned, and the CCFR retained those blocks for long-term storage and recuts. For the present study, FFPE were both newly cut, and some stored tissues were used. If we did not have sufficient tissue stored as slides, we made fresh sections from stored blocks. The CCFR has tissue stored both as curls/scrolls in tubes and as unstained slides. For this study, we only used colorectal tumor tissue from slides so we could macrodissect, as needed. For paired normal tissues, we sectioned freshly cut tissue into tubes (10 microns) since those did not need to be macrodissected. Thickness of stored slides were generally 4–10 (typically 5 or 7) microns thick; fresh cuts were always 7 microns thick. We used a variety of thicknesses and tissue both from freshly cut blocks and tissue stored on slides. As described in detail elsewhere [23] a separate DNA extraction process was utilized for the extraction of bacterial DNA, which we optimized for use with FFPE tissue.

#### Targeted assay for *F. nucleatum*

We employed a droplet digital PCR (ddPCR) assay to determine the presence and extent of *F. nucleatum* in these tissues [23] Specifically, we used multiplex ddPCR designed to quantify *F. nucleatum* and host genomic DNA using primers and probes specific to the nusG gene *F. nucleatum* and the eukaryotic housekeeping gene *SLCOA2A1* [6].This allowed us to normalize counts of *F. nucleatum* by levels of the eukaryotic housekeeping gene in that tissue type (i.e., *F. nucleatum* count divided by *SLCO2A1* count).

#### *F. nucleatum* presence and enrichment

Capturing both the presence and extent of *F. nucleatum* in both colorectal tumor tissue and paired normal colorectal tissue from the same study participant allowed us to define *F. nucleatum* status based not only on the presence of this bacterium in tumor tissue but also on the extent to which this bacterium was specifically enriched in tumor tissue relative to paired normal tissue [24]. Thus, we considered two operationalizations of *F. nucleatum* status: enrichment and presence. Enrichment was calculated as the continuous difference in normalized *F. nucleatum* counts in tumor vs. in matched normal tissue (where positive values indicated that the normalized count of *F. nucleatum* in tumor was higher than that in matched normal tissue). Counts were zero in both tumor and normal tissues or the same in both tissues for about 75% of participants so we categorized this variable into three groups for analyses: no difference, tumor > normal, and normal > tumor.

*F. nucleatum* presence in tumor was classified categorically as not present (0 counts detected), low (normalized *F. nucleatum* counts in tumor > 0 but less than the median level among those positive for *F. nucleatum*), or high (normalized *F. nucleatum* counts in tumor greater than or equal to the median), without regard to *F. nucleatum* status in matched normal tissue.

#### 16S rRNA gene sequencing and bioinformatic processing

Polymerase chain reaction (PCR) was performed using 16S rRNA gene V4 primers. Samples were processed in USEARCH-UNOISE3 (usearchv11.0.667_i86linux64) [25, 26] and QIIME 2 [27] Amplicon sequencing variants (ASVs) were filtered by abundance (with a threshold of 1e-5) as suggested by Prodan et al., 2020 [28]. Taxonomy was assigned using the classify-sklearn module in QIIME2 with the SILVA 138 taxonomy [29] Phylogeny was constructed using the align-to-tree-mafft-fasttree command in QIIME2. This used MAFFT [30] and fasttree2 [31]. Samples were decontaminated with SCRuB v0.0.1 [32] ASVs were further filtered by applying a level of detection filter at the genus level, with a threshold set to 0.0009. We reviewed the genera lists generated based on 1) passing the limit of detection (LOD) levels (*n* = 136) and 2) the subset that passed limit of quantitation (LOQ) (*n* = 60). Evaluation was based on current literature specifically on contaminants in low biomass samples [33] while also considering possible colon tumor pathogens found via a method less prone to contaminant issues [34]. Genera considered contaminants were marked for exclusion (*n* = 23). Genera that passed only the LOD level but have been found by others to be present in colon tumors were added to the genera to keep (n = 11) [33, 34]. The total number of genera kept for analysis was 48.

#### Tumor tissue alpha diversity

Alpha diversity was assessed using the Shannon index [35]. We assessed alpha diversity in tumor tissue categorized according to tertiles (low: 0.003–2.59, medium: 2.59–3.42, high: 3.42–5.89). In supplementary analyses, we also examined tumor tissue alpha diversity continuously and alpha diversity in paired normal colorectal tissue.

### Statistical analysis

We conducted regression analyses to examine the associations of epidemiologic risk factors with *F. nucleatum* enrichment or presence and with tumor tissue alpha diversity. Multinomial logistic regression models were fit separately for 1) demographics; 2) health behaviors and personal history; and 3) nSES. The reference outcomes in multinomial logistic regression models were “no difference in counts between tumor and normal tissue” in enrichment models and “not present” in presence models. Analyses for demographic and behavioral risk factors were adjusted for all other demographic and behavioral risk factors, respectively. Health behavior and nSES models were also adjusted for age and sex. Data missingness was highest for alcohol intake (30%), physical activity (27%), and nSES (25%). Missingness across other variables ranged from 0 to 12%. Multiple imputation was used to account for missingness in all epidemiologic risk factors [36, 37]. Iterative rounds of imputation (*n* = 100) were performed using the Amelia II package [38] in R version 4.1.2.28. Imputation models included all demographic and behavioral risk factors, diagnosis year, and time from diagnosis to enrollment into the PSCCC.

## Results

### *F. nucleatum* enrichment and presence

Descriptions of participants, counts of *F. nucleatum* in tumor and patient-matched normal tissue, and *F. nucleatum* enrichment data are shown in Table 1. Normalized counts of *F. nucleatum* were consistently lower in normal tissue than in tumor tissue across all epidemiologic risk factors. Normalized *F. nucleatum* counts in normal tissue ranged from 0.001 to 0.006, whereas normalized counts in tumor tissue ranged between 0.02 and 0.06. *F. nucleatum* counts were greater in tumor than normal tissues for 18.4% of samples and greater in normal vs. tumor tissues for 7.6% of samples.

**Table 1:** Normalized *F. nucleatum* counts in tumor and paired normal colorectal tissue according to epidemiologic risk factors for CRC patients (*n* = 898)

		*F. nucleatum* counts^1^		
Epidemiologic risk factors	N (%)	Normal tissue^2^ Mean (SD)	Tumor tissue Mean (SD)	Enrichment^3^ Mean (SD)
Total	898 (100%)	0.003 (0.027)	0.032 (0.242)	0.029 (0.243)
Sex				
Male	402 (45%)	0.002 (0.017)	0.022 (0.160)	0.020 (0.161)
Female	496 (55%)	0.004 (0.033)	0.041 (0.292)	0.037 (0.294)
Age (years)				
< 40	72 (8.0%)	0.002 (0.007)	0.021 (0.072)	0.019 (0.072)
40–49	220 (24%)	0.001 (0.009)	0.017 (0.105)	0.016 (0.103)
50–59	174 (19%)	0.004 (0.046)	0.022 (0.152)	0.017 (0.156)
60–69	275 (31%)	0.004 (0.021)	0.044 (0.328)	0.040 (0.327)
70–74	157 (17%)	0.006 (0.031)	0.052 (0.324)	0.047 (0.328)
Race^4^				
White	790 (88%)	0.003 (0.027)	0.034 (0.253)	0.030 (0.254)
Non-White	107 (12%)	0.004 (0.026)	0.026 (0.140)	0.022 (0.142)
Missing	1			
Education				
Less than high school	69 (8.7%)	0.010 (0.074)	0.015 (0.055)	0.005 (0.081)
High school	192 (24%)	0.004 (0.018)	0.065 (0.431)	0.062 (0.433)
Vocational/technical/some college	271 (34%)	0.003 (0.023)	0.017 (0.080)	0.014 (0.082)
College or higher	260 (33%)	0.001 (0.009)	0.036 (0.238)	0.034 (0.235)
Missing	106			
Vegetable intake^5^				
High	325 (37%)	0.003 (0.017)	0.040 (0.236)	0.037 (0.235)
Medium	340 (38%)	0.005 (0.039)	0.017 (0.098)	0.012 (0.102)
Low	225 (25%)	0.002 (0.016)	0.046 (0.374)	0.044 (0.375)
Missing	8			
Fruit intake^5^				
High	275 (31%)	0.003 (0.019)	0.035 (0.237)	0.032 (0.237)
Medium	251 (29%)	0.001 (0.004)	0.048 (0.351)	0.047 (0.351)
Low	353 (40%)	0.005 (0.037)	0.021 (0.134)	0.016 (0.137)
Missing	19			
Red meat intake^5^				
Low	161 (19%)	0.006 (0.049)	0.014 (0.057)	0.008 (0.066)
Medium	361 (42%)	0.004 (0.024)	0.026 (0.154)	0.022 (0.156)
High	333 (39%)	0.002 (0.015)	0.039 (0.311)	0.037 (0.311)
Missing	43			
Alcohol Intake^5^				
None	421 (67%)	0.003 (0.033)	0.030 (0.207)	0.026 (0.209)
Medium	105 (17%)	0.004 (0.016)	0.027 (0.142)	0.024 (0.134)
High	98 (16%)	0.001 (0.009)	0.077 (0.518)	0.076 (0.518)
Missing	274			
Physical activity^6^				
Low	478 (73%)	0.002 (0.014)	0.030 (0.264)	0.029 (0.264)
Medium	143 (22%)	0.007 (0.052)	0.066 (0.349)	0.059 (0.352)
High	31 (4.8%)	0.009 (0.038)	0.009 (0.030)	0.000 (0.041)
Missing	246			
Cigarette use				
Ever	512 (57%)	0.005 (0.036)	0.035 (0.274)	0.030 (0.276)
Never	386 (43%)	0.001 (0.007)	0.029 (0.193)	0.028 (0.192)
Ever used NSAID				
No	459 (52%)	0.003 (0.020)	0.024 (0.148)	0.021 (0.147)
Yes	432 (48%)	0.004 (0.034)	0.042 (0.314)	0.038 (0.316)
Missing	7			
CRC family history				
No	748 (83%)	0.004 (0.029)	0.033 (0.249)	0.030 (0.250)
Yes	150 (17%)	0.003 (0.020)	0.029 (0.205)	0.026 (0.206)
BMI (kg/m^2^)				
< 25	288 (32%)	0.004 (0.023)	0.031 (0.213)	0.028 (0.213)
25–29	347 (39%)	0.004 (0.036)	0.047 (0.328)	0.043 (0.329)
30 +	262 (29%)	0.002 (0.017)	0.015 (0.095)	0.013 (0.095)
Missing	1	1	1	1
Neighborhood SES^7^				
Q1	191 (25%)	0.002 (0.010)	0.013 (0.076)	0.011 (0.076)
Q2	190 (25%)	0.006 (0.048)	0.026 (0.173)	0.020 (0.178)
Q3	190 (25%)	0.003 (0.013)	0.050 (0.282)	0.048 (0.281)
Q4	190 (25%)	0.004 (0.024)	0.026 (0.160)	0.022 (0.161)
Missing	137 (25%)	137	137	137

*CRC* colorectal cancer, *BMI* body mass index, *F. nucleatum Fusobacterium nucleatum*, *kg* kilogram, *m* meter, *NSAID* non-steroidal anti-inflammatory drug, *SES* socioeconomic status

^1^A multiplex droplet digital PCR (ddPCR) assay was used to determine the extent of *F. nucleatum* in tissues normalized to the amount of eukaryotic DNA levels

^2^Paired normal colorectal tissues from the same patient

^3^Enrichment was defined as the continuous difference in normalized *F. nucleatum* counts in tumor vs. in paired normal colorectal tissue (where positive values indicated that the normalized count of *F. nucleatum* in tumor was higher than that in matched normal tissue)

^4^Race was categorized as white and because there were few non-White participants in the study. Non-White participants included the following racial and ethnic groups: American Indian/Alaska Native (2.0%), Asian (4.2%), Black (3.2%), Hispanic (1.7%), and Pacific Islander (0.4%)

^5^Participants were asked about average dietary intake via telephone interview from a time approximately two years prior to CRC diagnosis. Vegetable, fruit, and red meat intake were assessed by asking participants, “Would you please tell me how often per day, per week, or per month you ate the following foods?” For vegetables and fruits, intake was categorized as < 1 serving/day (low), 1 serving/day (medium), and > 1 than one serving/day (high). Red meat intake was categorized into tertiles < 0.28 servings/day (low), 0.28–0.57 servings/day (medium), and > 0.57 servings/day (high). Alcohol intake was assessed in each decade of life by first asking participants, “Since turning [age]…/Think back to the period when you were in your [age decade]. During that time… Did you ever drink any alcoholic beverages at least once a week for 6 months or longer?” and if yes, “How many alcoholic beverages a week did you typically drink when you consumed at least 1 drink a week for 6 months or longer?” We used the most recent decade of life to categorize alcohol intake, and categories were no drinks per week (none), 1–6 drink(s) per week (medium), and ≥ 7 drinks per week (high)

^6^During the baseline telephone interview, participants were asked to self-report detailed information on their recreational physical activity during defined age periods prior to diagnosis: ages 20–29, 30–49, and 50 + years [20] Questions covered different modes of activities (e.g., walking, jogging, running, bicycling, swimming, soccer, tennis, basketball, calisthenics), and the usual duration and frequency of each activity. Evaluation was limited to activities in which the patients were engaged for at least 30 min per week, for at least 3 continuous months. Standard metabolic equivalent of task score (METS) values were assigned to each activity [21] and multiplied by the number of hours per week engaged in that activity to derive METS-hours per week (METS-h/week). Physical activity during the age period of a participant’s CRC diagnosis (i.e., 20–29, 30–49, or 50 + years) was then summarized as average METS-h/week. For the present analysis, we categorized this variable as < 10 (low), 10- 50 (medium), and > 50 METS-h/week (high)

^7^nSES was assessed on the Census Block group level using participant’s residential address at time of CRC diagnosis using a composite variable with six data elements from the American Community Survey as previously described [22]. In the composite variable, lower scores indicated lower nSES

Female sex was associated with *F. nucleatum* enrichment in tumor tissue relative to normal tissue (odds ratio [OR] 1.58, 95% confidence interval [CI] 1.10, 2.27) (Table 2). Female sex was also associated with both low and high presence of *F. nucleatum* in CRC tissue relative to the absence of detectable *F. nucleatum* (OR 1.77, 95%, CI 1.11, 2.83 and OR 1.61, 95% CI 1.02, 2.54, respectively) (Table 3). *F. nucleatum* presence and enrichment did not differ across any other epidemiologic risk factors, including dietary patterns and nSES.

**Table 2:** Associations of epidemiologic risk factors with *F. nucleatum* enrichment for CRC patients (*n* = 898)

	*F. nucleatum* count normal > tumor (ref no difference) *n* = 51 (7.6%)			*F. nucleatum* count tumor > normal (ref no difference) *n* = 164 (18.3%)		
	OR	95% CI	p-value	OR	95% CI	p-value
Model 1 Demographic factors						
Female sex	1.20	(0.67, 2.17)	0.54	**1.58**	**(1.10, 2.27)**	**0.01**
Age < 40 years (ref 40–49 years)	1.58	(0.39, 6.37)	0.52	1.86	(0.96, 3.58)	0.06
Age 50–59 years (ref 40–49 years)	1.86	(0.69, 5.04)	0.22	0.99	(0.57, 1.74)	0.98
Age 60–69 years (ref 40–49 years)	2.22	(0.89, 5.53)	0.09	1.41	(0.87, 2.28)	0.16
Age 70–74 years (ref 40–49 years)	**2.84**	**(1.08, 7.48)**	**0.03**	1.26	(0.72, 2.21)	0.41
Non-white race	1.20	(0.49, 2.98)	0.69	1.29	(0.77, 2.17)	0.33
< HS education (ref HS)	0.76	(0.24, 2.43)	0.64	1.03	(0.52, 2.03)	0.93
Vocational/technical/some college education (ref HS)	0.66	(0.30, 1.47)	0.31	0.96	(0.60, 1.54)	0.87
College degree or higher education (ref HS)	0.70	(0.31, 1.56)	0.38	0.78	(0.47, 1.28)	0.32
Model 2. Health behaviors and personal history						
Medium vegetable intake (ref high)	0.60	(0.30, 1.22)	0.16	0.73	(0.47, 1.12)	0.15
Low vegetable intake (ref high)	0.40	(0.15, 1.05)	0.06	0.67	(0.39, 1.15)	0.14
Medium fruit intake (ref high)	1.09	(0.50, 2.38)	0.83	0.88	(0.54, 1.42)	0.60
Low fruit intake (ref high)	1.26	(0.53, 3.01)	0.60	1.19	(0.72, 1.95)	0.50
Medium red meat intake (ref low)	0.96	(0.43, 2.14)	0.92	0.80	(0.48, 1.32)	0.38
High red meat intake (ref low)	0.95	(0.41, 2.19)	0.90	1.28	(0.78, 2.12)	0.33
Medium alcohol intake (ref none)	1.21	(0.48, 3.06)	0.69	1.31	(0.75, 2.29)	0.33
High alcohol intake (ref none)	0.78	(0.26, 2.32)	0.65	1.06	(0.59, 1.92)	0.85
Ever used cigarettes	**0.45**	**(0.23, 0.88)**	**0.02**	0.94	(0.65, 1.36)	0.73
Medium physical activity (ref low)	1.68	(0.80, 3.56)	0.17	1.56	(0.99, 2.47)	0.06
High physical activity (ref low)	1.85	(0.51, 6.64)	0.35	1.65	(0.73, 3.71)	0.23
Ever used NSAIDs	0.71	(0.39, 1.31)	0.27	0.79	(0.55, 1.13)	0.20
CRC family history	1.21	(0.59, 2.52)	0.60	0.62	(0.37, 1.05)	0.07
Model 3. Neighborhood SES						
nSES Q1 (ref Q4)	0.93	(0.40, 2.16)	0.87	1.08	(0.61, 1.91)	0.80
nSES Q2 (ref Q4)	0.90	(0.39, 2.10)	0.81	1.36	(0.78, 2.38)	0.27
nSES Q3 (ref Q4)	0.71	(0.28, 1.80)	0.47	1.49	(0.86, 2.56)	0.15

*CI* confidence interval, *CRC* colorectal cancer, *F. nucleatum Fusobacterium nucleatum*, *HS* high school, *kg* kilogram, *m* meter, *NSAID* non-steroidal anti-inflammatory drug, *nSES* neighborhood socioeconomic status, *OR* odds ratio

^1^Model estimates were generated using multinomial logistic regression where the outcome was *F. nucleatum* enrichment, defined as the continuous difference in *F. nucleatum* abundance between patient-matched tumor and normal tissue samples and categorized into three groups: no difference (ref), tumor > normal, and normal > tumor. Three models were fit separately for 1) demographics, 2) health behaviors and personal history, and 3) nSES. Age 40–49 years was chosen as the reference group because the < 40 years group had a small sample size. High school education was chosen as the reference group because < high school had a small sample size

**Table 3:** Associations of epidemiologic risk factors with *F. nucleatum* presence for CRC patients (*n* = 898)

	Low *F. nucleatum* presence (ref no presence)^1^ *n* = 92 (10.2%)			High *F. nucleatum* presence (ref no presence)^1^ *n* = 93 (10.3%)			
	OR	95% CI	p-value	OR	95% CI	p-value	p-trend^2^
Model 1. Demographic factors							
Female sex	**1.77**	**(1.11, 2.83)**	**0.02**	**1.61**	**(1.02, 2.54)**	**0.04**	*
Age < 40 years (ref 40–49 years)	1.95	(0.84, 4.52)	0.12	1.63	(0.70, 3.82)	0.26	
Age 50–59 years (ref 40–49 years)	1.26	(0.62, 2.55)	0.52	0.97	(0.48, 1.98)	0.94	
Age 60–69 years (ref 40–49 years)	1.42	(0.75, 2.68)	0.28	1.33	(0.73, 2.44)	0.35	
Age 70–74 years (ref 40–49 years)	1.50	(0.74, 3.05)	0.27	1.07	(0.53, 2.19)	0.85	
Non-white race	1.52	(0.82, 2.82)	0.18	1.02	(0.51, 2.02)	0.96	
< HS education (ref HS)	0.90	(0.34, 2.37)	0.84	1.05	(0.46, 2.38)	0.90	
Vocational/technical/some college education (ref HS)	1.20	(0.65, 2.21)	0.56	0.73	(0.40, 1.34)	0.31	
College degree or higher education (ref HS)	0.84	(0.43, 1.64)	0.61	0.73	(0.40, 1.35)	0.32	
Model 2. Health behaviors and personal history							
Medium vegetable intake (ref high)	0.81	(0.47, 1.40)	0.45	0.76	(0.44, 1.31)	0.32	
Low vegetable intake (ref high)	0.67	(0.33, 1.34)	0.25	0.66	(0.33, 1.32)	0.24	
Medium fruit intake (ref high)	0.83	(0.45, 1.53)	0.55	0.75	(0.41, 1.37)	0.35	
Low fruit intake (ref high)	1.20	(0.64, 2.26)	0.56	0.96	(0.52, 1.79)	0.90	
Medium red meat intake (ref low)	0.73	(0.39, 1.37)	0.33	1.04	(0.54, 1.97)	0.92	
High red meat intake (ref low)	1.26	(0.68, 2.34)	0.46	1.28	(0.66, 2.47)	0.47	
Medium alcohol intake (ref none)	1.45	(0.73, 2.89)	0.29	1.34	(0.66, 2.74)	0.41	
High alcohol intake (ref none)	0.86	(0.38, 1.98)	0.73	1.38	(0.68, 2.83)	0.37	
Ever used cigarettes	0.90	(0.56, 1.44)	0.66	0.71	(0.44, 1.14)	0.16	
Medium physical activity (ref low)	1.41	(0.76, 2.60)	0.27	1.79	(1.02, 3.13)	0.04	*
High physical activity (ref low)	2.01	(0.78, 5.14)	0.15	1.54	(0.55, 4.31)	0.42	
Ever used NSAIDs	**0.52**	**(0.32, 0.84)**	**0.01**	0.99	(0.62, 1.57)	0.96	
CRC family history	0.85	(0.46, 1.58)	0.60	0.37	**(0.17, 0.80)**	**0.01**	*
Model 3. Neighborhood SES							
nSES Q1 (ref Q4)	1.21	(0.61, 2.43)	0.58	0.92	(0.44, 1.92)	0.82	
nSES Q2 (ref Q4)	1.08	(0.53, 2.22)	0.83	1.41	(0.73, 2.76)	0.31	
nSES Q3 (ref Q4)	1.39	(0.71, 2.73)	0.33	1.34	(0.68, 2.64)	0.39	

*CI* confidence interval, *CRC* colorectal cancer, *F. nucleatum Fusobacterium nucleatum*, *HS* high school, *kg* kilogram, *m* meter, *NSAID* non-steroidal anti-inflammatory drug, *nSES* neighborhood socioeconomic status, *OR* odds ratio

^1^Model estimates were generated using multinomial logistic regression where the outcome was *F. nucleatum* presence classified categorically as no presence (ref), low (*F. nucleatum* abundance > 0 but < median level among those positive for *F. nucleatum*), or high (abundance ≥ median). Three models were fit separately for 1) demographics, 2) health behaviors and personal history, and 3) nSES. Age 40–49 years was chosen as the reference group because the < 40 years group had a small sample size. High school education was chosen as the reference group because < high school had a small sample size

^2^Tests for trend across categories of the outcome were conducted using ordinal logistic regression. Statistically significant p-trend values (p < 0.05) are indicated with an asterisk

To examine the robustness of associations for sex and *F. nucleatum*, we conducted additional analyses to explore effect modification according to tumor site and stage by stratifying data to the most common levels of tumor site (proximal) and stage (regional, I–II) and then rerunning the associations between sex and *F. nucleatum*. In stratified analyses, female sex was still associated with low *F. nucleatum* presence in proximal colon tumors (OR 2.80, 95% CI 1.34, 5.67) and with high *F. nucleatum* presence in regional cancers (OR 1.97, 95% CI 1.06, 1.67). We also examined whether sex differences in tumor site could have confounded the association but adjusting for tumor site does not change the estimated association between sex and *F. nucleatum* presence (results not shown).

### Alpha diversity

Table 4 reports mean alpha diversity in tumor tissue and paired normal colorectal tissue according to epidemiologic risk factors. The mean alpha diversity in tumor tissue was 3.02 (standard deviation [SD] 1.16) and was 3.22 (SD 1.18) in normal tissue. Alpha diversity was consistently lower in tumor tissue relative to patient-matched normal colorectal tissue across all epidemiologic risk factors.

**Table 4:** Alpha diversity in tumor and paired normal colorectal tissue according to epidemiologic risk factors for CRC patients (*n* = 611)

Alpha diversity^1^				
Epidemiologic risk factors	N (%)	Normal tissue^2^ Mean (SD)	Tumor tissue Mean (SD)	Difference^3^ Mean (SD)
Total	611 (100%)	3.222 (1.176)	3.025 (1.159)	-0.196 (1.047)
Sex				
Male	284 (46%)	3.155 (1.228)	3.069 (1.170)	-0.086 (1.083)
Female	327 (54%)	3.280 (1.128)	2.988 (1.150)	-0.292 (1.007)
Age (years)				
< 40	49 (8.0%)	3.093 (0.784)	2.797 (0.769)	-0.296 (1.033)
40–49	121 (20%)	3.320 (1.098)	3.120 (1.213)	-0.200 (1.003)
50–59	123 (20%)	3.268 (1.346)	3.115 (1.332)	-0.154 (1.135)
60–69	211 (35%)	3.153 (1.186)	2.964 (1.154)	-0.189 (1.036)
70–74	107 (18%)	3.252 (1.190)	3.044 (1.032)	-0.208 (1.037)
Race^4^				
White	537 (88%)	3.226 (1.171)	3.050 (1.161)	-0.176 (1.028)
Non-White	73 (12%)	3.179 (1.223)	2.848 (1.145)	-0.331 (1.185)
Missing	1	1	1	1
Education				
Less than high school	48 (8.9%)	2.952 (1.271)	2.899 (0.978)	-0.053 (0.950)
High school	137 (26%)	3.280 (1.237)	2.940 (1.241)	-0.340 (1.032)
Vocational/technical/some college	179 (33%)	3.293 (1.036)	3.092 (1.126)	-0.201 (0.975)
College or higher	173 (32%)	3.244 (1.125)	3.132 (1.105)	-0.111 (1.127)
Missing	74	74	74	74
Vegetable intake^5^				
High	217 (36%)	3.346 (1.097)	3.122 (1.151)	-0.224 (0.986)
Medium	242 (40%)	3.165 (1.219)	2.998 (1.151)	-0.168 (1.073)
Low	146 (24%)	3.143 (1.205)	2.942 (1.187)	-0.202 (1.100)
Missing	6	6	6	6
Fruit intake^5^				
High	196 (33%)	3.282 (1.094)	3.086 (1.151)	-0.196 (0.999)
Medium	173 (29%)	3.246 (1.097)	2.966 (1.175)	-0.280 (1.054)
Low	231 (39%)	3.140 (1.290)	3.031 (1.171)	-0.109 (1.064)
Missing	11	11	11	11
Red meat intake^5^				
Low	106 (18%)	3.127 (1.074)	2.868 (1.148)	-0.260 (1.093)
Medium	248 (43%)	3.208 (1.142)	3.066 (1.137)	-0.142 (1.032)
High	227 (39%)	3.293 (1.232)	3.068 (1.165)	-0.225 (1.064)
Missing	30	30	30	30
Alcohol Intake^5^				
None	290 (68%)	3.161 (1.135)	3.005 (1.135)	-0.156 (1.018)
Medium	75 (18%)	3.205 (1.072)	3.018 (1.180)	-0.187 (1.034)
High	59 (14%)	3.422 (1.176)	3.017 (1.211)	-0.405 (0.998)
Missing	187	187	187	187
Physical activity^6^				
Low	323 (72%)	3.217 (1.153)	3.043 (1.164)	-0.174 (1.011)
Medium	99 (22%)	3.271 (0.986)	3.047 (0.988)	-0.224 (1.119)
High	25 (5.6%)	3.426 (1.139)	3.199 (1.061)	-0.226 (0.881)
Missing	164	164	164	164
Cigarette use				
Ever	345 (56%)	3.178 (1.201)	2.946 (1.164)	-0.232 (1.048)
Never	266 (44%)	3.279 (1.143)	3.130 (1.148)	-0.149 (1.047)
Ever used NSAID				
No	305 (50%)	3.219 (1.146)	2.953 (1.170)	-0.266 (1.072)
Yes	300 (50%)	3.208 (1.210)	3.097 (1.143)	-0.111 (1.015)
Missing	6	6	6	6
CRC family history				
No	507 (83%)	3.195 (1.166)	2.985 (1.181)	-0.209 (1.017)
Yes	104 (17%)	3.355 (1.222)	3.223 (1.028)	-0.132 (1.189)
BMI (kg/m^2^)				
< 25	192 (31%)	3.328 (1.082)	3.025 (1.130)	-0.303 (1.015)
25–29	235 (39%)	3.210 (1.197)	3.102 (1.157)	-0.108 (1.036)
30 +	183 (30%)	3.131 (1.241)	2.928 (1.193)	-0.203 (1.090)
Missing	1	1	1	1
Neighborhood SES^7^				
Q1	129 (25%)	3.141 (1.157)	2.882 (1.170)	-0.259 (0.996)
Q2	127 (25%)	3.234 (1.132)	3.051 (1.096)	-0.184 (1.024)
Q3	119 (23%)	3.243 (1.221)	3.173 (1.157)	-0.070 (1.010)
Q4	134 (26%)	3.371 (1.024)	3.155 (1.093)	-0.216 (1.082)
Missing	102	102	102	102

*CRC* colorectal cancer, *BMI* body mass index, *kg* kilogram, *m* meter, *NSAID* non-steroidal anti-inflammatory drug, *SES* socioeconomic status

^1^Alpha diversity was assessed using the Shannon index

^2^Paired normal colorectal tissues from the same patient

^3^Defined as the continuous difference in Shannon alpha diversity in tumor vs. in paired normal colorectal tissue (where positive values indicated that the Shannon alpha diversity in tumor was higher than that in matched normal tissue)

^4^Race was categorized as White and because there were few non-White participants in the study. Non-White participants included the following racial and ethnic groups: American Indian/Alaska Native (2.3%), Asian (4.1%), Black (3.8%), Hispanic (0.8%), and Pacific Islander (0.5%)

^5^Participants were asked about average dietary intake via telephone interview from a time approximately two years prior to CRC diagnosis. Vegetable, fruit, and red meat intake were assessed by asking participants, “Would you please tell me how often per day, per week, or per month you ate the following foods?” For vegetables and fruits, intake was categorized as < 1 serving/day (low), 1 serving/day (medium), and > 1 than one serving/day (high). Red meat intake was categorized into tertiles < 0.28 servings/day (low), 0.28–0.57 servings/day (medium), and > 0.57 servings/day (high). Alcohol intake was assessed in each decade of life by first asking participants, “Since turning [age]…/Think back to the period when you were in your [age decade]. During that time… Did you ever drink any alcoholic beverages at least once a week for 6 months or longer?” and if yes, “How many alcoholic beverages a week did you typically drink when you consumed at least 1 drink a week for 6 months or longer?” We used the most recent decade of life to categorize alcohol intake, and categories were no drinks per week (none), 1–6 drink(s) per week (medium), and ≥ 7 drinks per week (high)

^6^During the baseline telephone interview, participants were asked to self-report detailed information on their recreational physical activity during defined age periods prior to diagnosis: ages 20–29, 30–49, and 50 + years [20]. Questions covered different modes of activities (e.g., walking, jogging, running, bicycling, swimming, soccer, tennis, basketball, calisthenics), and the usual duration and frequency of each activity. Evaluation was limited to activities in which the patients were engaged for at least 30 min per week, for at least 3 continuous months. Standard metabolic equivalent of task score (METS) values were assigned to each activity [21] and multiplied by the number of hours per week engaged in that activity to derive METS-hours per week (METS-h/week). Physical activity during the age period of a participant’s CRC diagnosis (i.e., 20–29, 30–49, or 50 + years) was then summarized as average METS-h/week. For the present analysis, we categorized this variable as < 10 (low), 10- 50 (medium), and > 50 METS-h/week (high)

^7^Neighborhood SES was assessed on the Census Block group level using participants’ residential address at time of CRC diagnosis using a composite variable with six data elements from the American Community Survey as previously described [22]. In the composite variable, lower scores indicated lower neighborhood SES

Relative to those aged 40–49 years, those in the youngest age group (< 40 years) had lower alpha diversity (OR for highest vs. lowest tertile: 0.33; 95% 0.13, 0.83 (Table 5). Alpha diversity in tumor tissue was greater among those reporting a family history of CRC (OR 1.92; 95% CI 1.10, 3.33). Alpha diversity in tumor tissue did not differ across any aspects of diet, including vegetable, fruit, red meat, and alcohol intake. There were no statistically significant associations between epidemiologic risk factors and alpha diversity in normal tissue (**Supplementary Material**).

**Table 5:** Association of epidemiologic risk factors with tertile of tumor tissue alpha diversity (*n* = 611)

	Outcome: Medium tumor tissue alpha diversity (ref low)^1^ N = 203 (33.3%)			Outcome: High tumor tissue alpha diversity (ref low)^1^ N = 204 (33.4%)		
	OR	95% CI	p-value	OR	95% CI	p-value
Model 1. Demographic factors						
Female sex	0.94	(0.63, 1.39)	0.75	0.92	(0.62, 1.37)	0.69
Age < 40 years (ref 40–49 years)	0.86	(0.40, 1.87)	0.71	**0.33**	**(0.13, 0.83)**	**0.02**
Age 50–59 years (ref 40–49 years)	0.83	(0.44, 1.59)	0.58	1.03	(0.56, 1.92)	0.92
Age 60–69 years (ref 40–49 years)	0.79	(0.45, 1.39)	0.41	0.71	(0.41, 1.25)	0.24
Age 70–74 years (ref 40–49 years)	0.86	(0.45, 1.65)	0.65	0.67	(0.35, 1.30)	0.24
Non-white race	0.99	(0.55, 1.78)	0.98	0.66	(0.35, 1.24)	0.19
< HS education (ref HS)	0.83	(0.39, 1.77)	0.63	0.72	(0.31, 1.68)	0.44
Vocational/technical/some college education (ref HS)	1.15	(0.67, 1.97)	0.62	1.24	(0.71, 2.17)	0.45
College degree or higher education (ref HS)	0.82	(0.47, 1.43)	0.48	1.24	(0.71, 2.15)	0.46
Model 2. Health behaviors and personal history						
Medium vegetable intake (ref high)	1.13	(0.69, 1.84)	0.64	0.97	(0.59, 1.60)	0.90
Low vegetable intake (ref high)	0.99	(0.53, 1.83)	0.97	0.85	(0.45, 1.60)	0.62
Medium fruit intake (ref high)	0.71	(0.42, 1.22)	0.21	0.67	(0.39, 1.15)	0.15
Low fruit intake (ref high)	0.90	(0.51, 1.61)	0.73	0.95	(0.53, 1.71)	0.87
Medium red meat intake (ref low)	1.44	(0.82, 2.53)	0.20	1.39	(0.78, 2.46)	0.26
High red meat intake (ref low)	1.28	(0.71, 2.30)	0.40	1.37	(0.76, 2.47)	0.30
Medium alcohol intake (ref none)	0.76	(0.39, 1.49)	0.43	1.27	(0.67, 2.40)	0.46
High alcohol intake (ref none)	0.96	(0.49, 1.88)	0.91	1.03	(0.51, 2.05)	0.94
Never used cigarettes	1.05	(0.69, 1.61)	0.81	**1.56**	**(1.02, 2.39)**	**0.04**
Low physical activity (ref high)	0.72	(0.29, 1.81)	0.49	1.05	(0.38, 2.95)	0.92
Medium physical activity (ref high)	0.79	(0.28, 2.23)	0.65	1.05	(0.34, 3.25)	0.93
Ever used NSAIDs	1.35	(0.89, 2.03)	0.15	1.43	(0.95, 2.17)	0.09
CRC family history	1.21	(0.68, 2.16)	0.52	**1.94**	**(1.12, 3.37)**	**0.02**
Model 3. Neighborhood SES						
nSES Q1 (ref Q4)	1.01	(0.56, 1.79)	0.98	0.69	(0.38, 1.28)	0.24
nSES Q2 (ref Q4)	0.84	(0.46, 1.52)	0.56	0.81	(0.45, 1.47)	0.49
nSES Q3 (ref Q4)	0.97	(0.52, 1.80)	0.92	1.16	(0.64, 2.11)	0.63

*CI* confidence interval, *CRC* colorectal cancer, *HS* high school, *kg* kilogram, *m* meter, *NSAID* non-steroidal anti-inflammatory drug, *nSES* neighborhood socioeconomic status, *OR* odds ratio

^1^Alpha diversity was assessed using the Shannon index [35] and categorized according to tertiles (low: 0.003–2.59, medium: 2.59–3.42, high: 3.42–5.89). Model estimates were generated using multinomial logistic regression. Three models were fit separately for 1) demographics, 2) health behaviors and personal history, and 3) nSES. Age 40–49 years was chosen as the reference group because the < 40 years group had a small sample size. High school education was chosen as the reference group because < high school had a small sample size

## Discussion

In this study, we explored whether established epidemiologic risk factors for CRC were associated with the presence and enrichment of *F. nucleatum* and with bacterial diversity in colorectal tumor tissue. *F. nucleatum*, a bacterial species found in the gut of CRC patients and linked to CRC outcomes [8] was more commonly present in tumor tissue than in patient-matched normal colorectal tissue and female sex was associated with both presence and enrichment of *F. nucleatum* in tumor tissue. However, our findings did not provide evidence that other epidemiologic risk factors for CRC, including age, aspects of diet, or nSES, were associated with *F. nucleatum* presence or absence in tumors. We did observe greater alpha diversity in patient-matched normal colorectal tissue relative to tumor tissue, with some indication that bacterial alpha diversity in tumor tissue differed across certain epidemiology characteristics including age.

Enrichment of *F. nucleatum* in invasive colorectal tumor tissues relative to normal colorectal tissue has been documented in multiple prior studies [6, 39, 40]. Studies have also demonstrated that microbial diversity in the gut is lower among CRC patients than in adenoma samples or samples from healthy controls [1, 12, 41]. *F. nucleatum* is likely overrepresented in tumor tissues due to its ability to bind to and invade cancer cells via adhesion proteins and because of the mechanisms by which it promotes cancer progression through increased expression of oncogenes, transcription factors, inflammatory genes, and Wnt signaling [5, 6, 10]. Similarly, it is these conditions in the tumor microenvironment that select specific bacteria and lessen the richness of bacterial species. By evaluating characteristics of not only tumor tissue but also patient-matched normal tissue, our study was able to evaluate the role of epidemiologic factors in bacterial diversity localized to tumor regions and more diffusely beyond the tumor margins. However, it remains unclear whether these observed patterns of *F. nucleatum* presence and bacterial diversity precede the development of CRC or occur as a result.

We did not detect differences in *F. nucleatum* or alpha diversity in tumor tissue according to dietary variables, including vegetable, fruit, red meat, and alcohol intake. Although at least one other study similarly reported that the presence of *Fusobacterium* genus bacteria in fecal samples from patients with CRC was not associated with intake of meat (any, red, and processed), vegetables, whole grains, or alcohol [42] this null finding is largely inconsistent with previous work reporting associations of diet with aspects of the intratumoral microbiota. For instance, intake of specific dietary components like dairy [43] and the variety and vegetables and fruits [44] as well as overall dietary patterns characterized as inflammatory [14] or as fiber rich [15] have been linked to the presence and level of *F. nucleatum* in fecal samples from healthy adults and in colorectal tumors. Analyses of studies with fewer participants have also reported that intestinal abundance of *F. nucleatum* [45] and overall gut microbiota composition [46] statistically significantly mediate the relationship between dietary intake and CRC risk. Characteristics of the gut microbiota may play a mediating role in associations between the established CRC risk factor diet and CRC risk and/or survival and it is not clear why our findings deviate from the current literature. One reason could be related to our assessment of tumor tissue while previous work has examined the fecal microbiome. It is also possible there were limitations in our ascertainment of diet history, including measurement in a period not relevant for the composition of gut microbial composition. Further, our measures may not have wholly captured the most relevant nutrients impacting the gut microbiota (fiber, dairy, probiotics, etc.).

We found that female CRC patients had greater presence of *F. nucleatum* in tumor tissue than males. One prior study reported that among 1,069 CRC patients, high levels of *F. nucleatum* DNA in CRC tissue was more common in women than men, although differences were not statistically significant [8]. Work examining epidemiologic factors beyond diet in relation to gut *F. nucleatum* are limited, but at least some work has suggested cigarette smoking could be related to the presence of *F. nucleatum* in other anatomic sites like the mouth [47]. Further, a pilot study of 30 individuals with CRC showed that the *Fusobacteriota* phylum was more abundant in fecal samples among Black participants relative to participants of other races [48]. However, we did not detect differences in presence or enrichment of *F. nucleatum* or alpha diversity according to cigarette smoking or race.

Lastly, we found that the youngest participants had the lowest bacterial alpha diversity in tumor tissue. Other work has examined tumor bacterial diversity across young-onset and average onset cancers, although it remains equivocal whether lower or greater alpha diversity is more influential for CRC diagnosis [16, 17]. With the rising incidence of young-onset CRC, the role of the gut microbiota is an active area of research.

A better understanding of the role of epidemiologic risk factors in shaping the tumor-associated microbiota will allow for targeted CRC prevention and detection strategies. Several modifiable risk factors, including diet, physical activity, smoking, and use of medications like NSAIDS, sex hormones, supplements, antibiotics, and prebiotics could be targeted to promote greater bacterial diversity in the gut and to reduce the colonization of pathogenic species like *F. nucleatum*. Further detailed investigations into the risk factors likely to shape the microbial community in the gut and in the tumor of CRC patients along with quantification of microbial pathways and metabolites are needed for translational work.

## Strengths and limitations

This study is strengthened by the population-based sample with a wide age range, including both males and females for which normal and tumor tissue samples were available for comparison analyses. In addition, we were able to examine the impact of contextual (environmental) risk factors like nSES. Few studies have been able to examine the associations of epidemiologic risk factors for CRC and the tumor-associated gut microbiota to the extent for which we have available data. However, our study also has limitations. The intratumoral and normal tissue microbiota were assessed at one point in time which limits the ability to detect changes in the gut microbiota and the temporal sequence between epidemiologic risk factors, changes in gut microbiota composition, and CRC risk and progression. Further, epidemiologic data may have been ascertained at timepoints that are not relevant to assessment of the gut microbiota. Lastly, the study population consisted primarily of white non-Hispanic participants which precludes investigation into bacterial differences across race and ethnicity and limits generalizability of findings. Preliminary analyses in other work have suggested that race is an important factor for the gut microbiota and some racial and ethnic groups experience disproportionate burdens of CRC so additional analyses into these relationships are warranted for racially and ethnically diverse populations.

In conclusion, using colorectal tumor and paired normal colorectal tissue samples from a subset of participants in the population-based PSCCC, we found the presence of *F. nucleatum* and tumor tissue alpha diversity to differ across age and sex. In comparison, we found no evidence for associations between aspects of diet and gut bacterial characteristics. By identifying potential epidemiologic risk factors linked to aspects of the intratumoral microbiota, this work contributes to efforts to understand the role of the gut microbiota composition in the etiology and progression of CRC.

## Supplementary Information

Below is the link to the electronic supplementary material.

Supplementary file1 (DOCX 293 KB)

## Data Availability

The Colon CFR (CCFR) investigators and institutions affirm their intention to share research data consistent with all relevant NIH resource/data sharing policies. The CCFR (https://coloncfr.org/for-researchers/collaborate-with-the-ccfr/](https:/coloncfr.org/for-researchers/collaborate-with-the-ccfr). Microbial data reported in this manuscript can be requested by contacting the corresponding author and submitting an Application for Collaboration to the CCFR. Microbial data can be made available upon reasonable request to the corresponding author (A.I.P.).
