# Supplementary material for: Associations of epidemiologic risk factors with Fusobacterium nucleatum and bacterial alpha diversity in the colorectal tumor-associated microbiota
Source: Cancer Causes Control. 2026 Feb 10;37(3):45. doi: 10.1007/s10552-026-02133-4 (PMC12891059; doi:10.1007/s10552-026-02133-4)
Supplement: Supplementary file 1 — Supplementary file1 (DOCX 293 KB) [file 10552_2026_2133_MOESM1_ESM.docx]

**SUPPLEMENTARY MATERIAL**

**Associations of epidemiologic risk factors with *Fusobacterium nucleatum* and bacterial alpha diversity in the colorectal tumor-associated microbiota**

**Running title**

Epidemiologic risk factors for tumor-associated microbiota

**
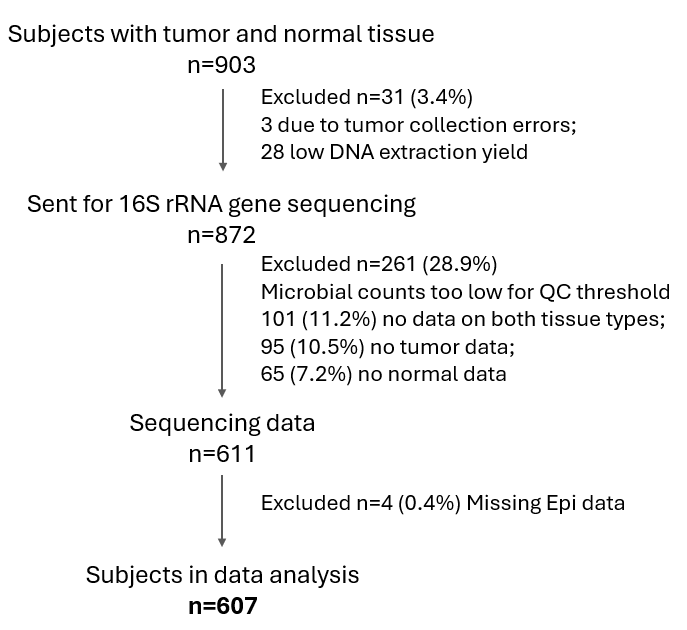
**

**Supplementary Figure 1. Diagram showing loss of samples for the 16S rRNA gene sequencing analysis**

**Supplementary Table 1. Association of epidemiologic risk factors with continuous tumor tissue alpha diversity**

|  | Outcome: Tumor tissue alpha diversity | | |
| --- | --- | --- | --- |
|  | Beta | 95% CI | p-value |
| **Model 1. Demographic factors** |  |  |  |
| Female sex | -0.07 | (-0.26, 0.12) | 0.47 |
| Age <40 years (ref 40-49 years) | -0.32 | (-0.72, 0.07) | 0.11 |
| Age 50-59 years (ref 40-49 years) | -0.01 | (-0.30, 0.29) | 0.97 |
| Age 60-69 years (ref 40-49 years) | -0.16 | (-0.42, 0.11) | 0.25 |
| Age 70-74 years (ref 40-49 years) | -0.05 | (-0.36, 0.25) | 0.73 |
| Non-white race | -0.20 | (-0.49, 0.09) | 0.17 |
| <HS education (ref HS) | -0.02 | (-0.41, 0.37) | 0.91 |
| Vocational/technical/some college education (ref HS) | 0.15 | (-0.12, 0.42) | 0.27 |
| College degree or higher education (ref HS) | 0.18 | (-0.09, 0.45) | 0.19 |
|  |  |  |  |
| **Model 2. Health behaviors and personal history** |  |  |  |
| Medium vegetable intake (ref high) | -0.15 | (-0.38, 0.08) | 0.19 |
| Low vegetable intake (ref high) | -0.25 | (-0.53, 0.04) | 0.10 |
| Medium fruit intake (ref high) | -0.05 | (-0.30, 0.20) | 0.70 |
| Low fruit intake (ref high) | 0.05 | (-0.21, 0.32) | 0.69 |
| Medium red meat intake (ref low) | 0.20 | (-0.07, 0.46) | 0.15 |
| High red meat intake (ref low) | 0.16 | (-0.11, 0.44) | 0.25 |
| Medium alcohol intake (ref none) | 0.04 | (-0.26, 0.33) | 0.80 |
| High alcohol intake (ref none) | 0.01 | (-0.30, 0.33) | 0.93 |
| Never used cigarettes | **0.23** | **(0.03, 0.42)** | **0.02** |
| Low physical activity (ref high) | -0.09 | (-0.53, 0.34) | 0.68 |
| Medium physical activity (ref high) | -0.10 | (-0.58, 0.38) | 0.68 |
| Ever used NSAIDs | 0.13 | (-0.06, 0.32) | 0.18 |
| CRC family history | 0.22 | (-0.03, 0.47) | 0.08 |
|  |  |  |  |
| **Model 3. Neighborhood SES** |  |  |  |
| NSES Q1 (ref Q4) | -0.21 | (-0.49, 0.07) | 0.15 |
| NSES Q2 (ref Q4) | -0.08 | (-0.37, 0.20) | 0.56 |
| NSES Q3 (ref Q4) | 0.00 | (-0.29, 0.29) | 0.99 |

CI, confidence interval. CRC, colorectal cancer; HS, high school; kg, kilogram; m, meter; NSAID, non-steroidal anti-inflammatory drug; nSES, neighborhood socioeconomic status; OR, odds ratio.

**Supplementary Table 2. Association of epidemiologic risk factors with paired normal tissue alpha diversity**

|  | Outcome: Medium normal tissue alpha diversity (ref low)^1^ | | | Outcome: High normal tissue alpha diversity (ref low)^1^ | | |
| --- | --- | --- | --- | --- | --- | --- |
|  | OR | 95% CI | p-value | OR | 95% CI | p-value |
| **Model 1. Demographic factors** | | | | | | |
| Female sex |  |  |  |  |  |  |
| Age <40 years (ref 40-49 years) | 1.57 | (1.05, 2.34) | 0.03 | 1.16 | (0.78, 1.73) | 0.46 |
| Age 50-59 years (ref 40-49 years) | 1.44 | (0.64, 3.25) | 0.38 | 0.69 | (0.28, 1.72) | 0.43 |
| Age 60-69 years (ref 40-49 years) | 0.77 | (0.41, 1.46) | 0.42 | 0.95 | (0.51, 1.75) | 0.86 |
| Age 70-74 years (ref 40-49 years) | 0.90 | (0.52, 1.59) | 0.73 | 0.69 | (0.39, 1.22) | 0.21 |
| Non-white race | 0.67 | (0.35, 1.30) | 0.24 | 0.77 | (0.40, 1.46) | 0.42 |
| <HS education (ref HS) | 0.80 | (0.44, 1.46) | 0.47 | 0.78 | (0.42, 1.43) | 0.42 |
| Vocational/technical/some college education (ref HS) | 1.11 | (0.52, 2.39) | 0.78 | 0.50 | (0.21, 1.22) | 0.13 |
| College degree or higher education (ref HS) | 0.96 | (0.55, 1.68) | 0.88 | 0.83 | (0.48, 1.45) | 0.51 |
|  |  |  |  |  |  |  |
| **Model 2. Health behaviors and personal history** | | | | | | |
| Medium vegetable intake (ref high) | 1.18 | (0.71, 1.96) | 0.52 | 0.83 | (0.51, 1.36) | 0.46 |
| Low vegetable intake (ref high) | 1.84 | (0.97, 3.49) | 0.06 | 0.96 | (0.51, 1.81) | 0.91 |
| Medium fruit intake (ref high) | 0.72 | (0.42, 1.24) | 0.23 | 1.11 | (0.64, 1.91) | 0.72 |
| Low fruit intake (ref high) | 0.53 | (0.30, 0.95) | 0.03 | 0.98 | (0.55, 1.74) | 0.94 |
| Medium red meat intake (ref low) | 1.10 | (0.63, 1.93) | 0.74 | 1.16 | (0.65, 2.08) | 0.62 |
| High red meat intake (ref low) | 1.05 | (0.58, 1.90) | 0.86 | 1.43 | (0.79, 2.60) | 0.24 |
| Medium alcohol intake (ref none) | 1.53 | (0.79, 2.98) | 0.20 | 1.31 | (0.68, 2.53) | 0.42 |
| High alcohol intake (ref none) | 1.55 | (0.79, 3.07) | 0.20 | 1.40 | (0.71, 2.77) | 0.33 |
| Never used cigarettes | 1.21 | (0.79, 1.85) | 0.38 | 1.17 | (0.77, 1.79) | 0.46 |
| Low physical activity (ref high) | 0.62 | (0.22, 1.71) | 0.35 | 0.70 | (0.27, 1.85) | 0.47 |
| Medium physical activity (ref high) | 0.81 | (0.27, 2.37) | 0.70 | 0.76 | (0.27, 2.15) | 0.60 |
| Ever used NSAIDs | 1.28 | (0.85, 1.93) | 0.24 | 1.13 | (0.75, 1.69) | 0.56 |
| CRC family history | 0.94 | (0.54, 1.65) | 0.83 | 1.20 | (0.70, 2.05) | 0.51 |
|  |  |  |  |  |  |  |
| **Model 3. Neighborhood SES** | | | | | | |
| NSES Q1 (ref Q4) | 1.16 | (0.64, 2.10) | 0.62 | 0.75 | (0.41, 1.37) | 0.35 |
| NSES Q2 (ref Q4) | 1.02 | (0.56, 1.87) | 0.95 | 0.94 | (0.52, 1.69) | 0.84 |
| NSES Q3 (ref Q4) | 0.97 | (0.53, 1.79) | 0.92 | 1.01 | (0.56, 1.82) | 0.97 |

CI, confidence interval. CRC, colorectal cancer; HS, high school; kg, kilogram; m, meter; NSAID, non-steroidal anti-inflammatory drug; nSES, neighborhood socioeconomic status; OR, odds ratio.
